# Androstenedione changes steroidogenic activity of SGBS cells

**DOI:** 10.1530/EC-19-0549

**Published:** 2020-06-03

**Authors:** Jana Ernst, Katharina Gert, Frank Bernhard Kraus, Ulrike Elisabeth Rolle-Kampczyk, Martin Wabitsch, Faramarz Dehghani, Kristina Schaedlich

**Affiliations:** 1 Department of Anatomy and Cell Biology, Faculty of Medicine, Martin Luther University Halle-Wittenberg, Grosse Steinstrasse, Halle (Saale), Germany; 2 Central Laboratory, University Hospital Halle (Saale), Ernst-Grube-Strasse, Halle (Saale), Germany; 3 Department of Molecular Systems Biology, Helmholtz Centre for Environmental Research Leipzig, Leipzig, Germany; 4 Division of Pediatric Endocrinology and Diabetes, Department of Pediatrics and Adolescent Medicine, University Medical Center Ulm, Ulm, Germany

**Keywords:** adipocytes, androgen, SGBS, steroid hormones

## Abstract

The rapid increase of obesity during the last decades and its future prospects are alarming. Besides the general discussed causes of obesity, the ‘Developmental Origins of Health and Disease’ (DOHaD) hypothesis received more attention in recent years. This hypothesis postulates an adverse influence during early development that programs the unborn child for metabolic dysfunctions later in life. Childhood obesity – an as much increasing problem – can be predisposed by maternal overweight and diabetes. Both, obesity and hyperinsulinemia are major causes of female hyperandrogenemia. As predicted by the DOHaD hypothesis and shown in animal models, developmental androgen excess can lead to metabolic abnormalities in offspring. In this study, we investigated, if androgen exposure adversely affects the adipogenic differentiation of preadipocytes and the endocrine function of adult adipocytes. The human SGBS preadipocyte model was used to affirm the *de novo* biosynthesis of steroid hormones under normal adipogenesis conditions. Normal adipogenesis was paralleled by an increase of corticosteroids and androgens, whereas estrogen remained at a steady level. Treatment with androstenedione had no effect on SGBS proliferation and differentiation, but adult adipocytes exhibited a significant higher accumulation of triglycerides. Progesterone (up to 2-fold), testosterone (up to 38-fold) and cortisone (up to 1.4-fold) – but not cortisol – were elevated by androstenedione administration in adult adipocytes. Estrogen was not altered. Data suggest that androgen does not negatively influence adipogenic differentiation, but steroidogenic function of SGBS adipocytes.

## Introduction

Across the OECD area more than one in five adults and nearly one in six children are overweight (BMI ≥ 25 kg/m^2^) or obese (BMI ≥ 30 kg/m^2^), and a steady increase in obesity rates until 2030 is expected (1). Obesity and its comorbidities are symptoms of the metabolic syndrome, that currently is one of the main social and public health problems. In fact, the worldwide epidemic of overweight and obesity, its rapid increase over the last decades and its future prospects has been termed as ‘globesity’ by the World Health Organization (WHO) (2). In causal research the ‘Developmental Origins of Health and Disease’ (DOHaD) hypothesis has attracted more and more attention during the last years. It postulates that an adverse influence during early development (i.e. preconceptional up to 1000 days of postnatal life) can program the unborn/newborn for metabolic dysfunctions later in life. Apart from environmental factors maternal diseases such as overweight or excessive weight gain during pregnancy and diabetes (type 1, type 2 or gestational) are among the adverse influences put forward by this hypothesis (3, 4, 5).

Obesity and hyperinsulinemia are major causes for female hyperandrogenemia. The thereby elevated androgen levels in the circulation result from an exceeding and dysregulated production of androgens by classical steroidogenic organs (6, 7). Classical steroidogenic organs such as the adrenal gland and the gonads are characterized by the presence of an enzyme machinery enabling them for the *de novo* synthesis of steroid hormones from cholesterol (8). Androgenic steroids exhibit a positive gradient from blood to adipose tissue with the precursor androstenedione and dehydroepiandrosterone as well as the active androgen testosterone, being the most abundant in the circulation (9). However, it has also been hypothesized that a further and substantial contributor to androgen production is adipose tissue (10). Besides the body’s own endocrine dysregulations, an environmental exposure to androgens exists due to the prevalent contamination of surface water and groundwater (11, 12, 13). Androgen excess during early development is hypothesized to program metabolic abnormalities in male and female offspring as demonstrated in mice (14, 15, 16) and rhesus monkeys (17, 18, 19) as wells as in humans (17, 18, 19, 20, 21).

Changes in the levels of circulating steroid hormones influence the steroidogenic activity of the adipose tissue and may result in an adipose tissue dysfunction with abnormal adipokine synthesis and disturbance of the lipid homeostasis and adipogenesis (22). Synthesis and metabolism of steroid hormones are regulated by a complex network of steroidogenic enzymes. Studies on this topic so far showed partly contradictory results due to investigations on isolated aspects of the network, differences between species and sexes as well as specificities of fat depots (21, 22, 23, 24).

Only a few studies have dealt with the impact of androgens on steroid hormone synthesis in human adipose tissue so far. Wabitsch and colleagues could show that human Simpson-Golabi-Behmel syndrome (SGBS) preadipocytes are comparable to *in vitro* matured adipocytes from human s.c. fat and as such are a good model system (25, 26). By demonstrating the conversion of cholesterol to pregnenolone as the initial step of steroid biosynthesis in the SGBS model, the evidence for adipocytes as steroidogenic cells was established as well (27). Additionally, the ability to secrete adipokines like leptin and adiponectin (28, 29, 30) provides it for the investigation of the endocrine function of (pre)adipocytes during adipogenesis and for the characterization of interactions of endocrine signaling. In the present study, we studied the steroidogenic activity of SGBS adipocytes followed by the investigation of the impact of androstenedione as a precursor of active steroid hormones on adipogenesis, steroid hormone synthesis and the adipokine system.

## Material and methods

### Cell culture

SGBS preadipocytes, a non-immortalized cell model for adipogenic differentiation, were cultured and differentiated to mature adipocytes as described earlier (25, 26). Culture medium was changed at day 4 of adipogenesis replacing the induction medium by the differentiation medium. Application of 10 µM 4-androstene-3,17-dione (Sigma-Aldrich) occurred from day 0 to day 8 of adipogenesis. Samples for mRNA expression and hormone measurement were taken at day 0, 2, 4, 6 and 8.

### Western blot analyses

To investigate the possible effects of androstenedione on cell proliferation, SGBS preadipocytes were cultured in basal medium for 2 days before adipogenic induction (26). Thereafter, androstenedione was applied for 24 h. For Western blot analyses, the SGBS cells were harvested and lysed in RIPA buffer containing 10x PBS, Nonidet NP40, 10% SDS, 0.5% sodiumdeoxycholate, protease and phosphatase inhibitors (Roche). Separation of total protein lysates by SDS-PAGE and the transfer to nitrocellulose membranes were performed. Blots were blocked in 0.1% TBST with 3% (wt/vol) BSA for 2 h. The primary antibodies against proliferating-cell-nuclear-antigen (PCNA) (1:1000; Cell Signaling) and β-ACTIN (1:10,000; Sigma Aldrich) were incubated at 4°C overnight. Afterwards, the secondary horseradish peroxidase-conjugated antibody goat anti-mouse (1:20,000; Dianova, Hamburg, Germany) was applied for 1 h at room temperature.

For the immunodetection of proteins at d8 of adipogenesis the procedure of cell lysis, protein separation and blot preparation was performed as described above. The primary antibodies against HSL (1:500; Cell Signaling), pHSL (1:250; Cell Signaling) and Perilipin (1:500; Cell Signaling) were incubated at 4°C overnight. Afterwards the secondary horseradish peroxidase (HRP)-conjugated antibodies goat anti-mouse (1:10,000; Dianova) or goat anti-rabbit (1:6000; Dako) were applied for 1 h at room temperature. The primary antibody against GAPDH (1:1000; Cell Signaling) was already conjugated to horseradish peroxidase and did not require a secondary antibody. Detection was performed with the immobilon western chemiluminescent HRP substrate (MerckMillipore). Subsequently, for analyses the ChemiDoc™ Touch MP Imaging System (BioRad) and the Image Lab 6.0 software were used. The protein amount was calculated as the ratio of each protein vs β-ACTIN or GAPDH intensity.

### Quantitative real-time PCR

Quantitative real-time PCR (qRT-PCR) was carried out on a StepOnePlus™ real-time PCR system (Applied Biosystems) to measure mRNA expression levels in the SGBS preadipocytes. Transcription with normalisation to 10^3^ copies of the TATA-box-binding protein (*TBP*) as housekeeping gene was determined for the following genes: *AR*, *CYP11A1*, *CYP17A1*, *CYP19, ER#x03B1;*, *ERβ*, *GLUT4*, *GPER*, *GR*, *HSD3B*,* HSD11B1*, *HSD17B5*, *PGR* and *StAR*. The specifications of primers and amplicons are given in Table 1. Quantitative standard curves were generated for each gene using a plasmid dilution series containing the target sequences (10^3^–10^6^ copies).

**Table 1:** Primers for quantitative RT-PCR.

Gene	Accession number	Forward primer	Reverse primer	Tm (°C)	Amplicon (bp)
*AR*	NM_000044	AATTCCTGTGCATGAAAGCCA	AGCTCTCTCGCAATAGGCTG	58	211
*CYP19*	NM_000103	ATGTGGACGTGTTGACCCTTCT	AGGAGAGCTTGCCATGCATCAA	60	133
*CYP11A1*	NM_000781	CTCAGTCCTGGTCAAAGGCT	CTTCTCCCTGTAAATCGGGC	63	250
*ERα*	NM_000125	CAATGACTATGCTTCAGGCTAC	CCACCTTTCATCATTCCCAC	60	198
*ERβ*	NM_001437	AGCCACCATGAATATCCAGCCA	TGGCCACAACACATTTGGGCTT	60	129
*GLUT4*	NM_001042	ACTGGCCATTGTTATCGGCA	GTCAGGCGCTTCAGACTCTT	60	213
*GPER*	NM_001505	ATGACCATCCCCGACCTGTA	GAGGAAGAAGACGCTGCTGT	60	174
*GR*	NM_000176	ACTGCTTCTCTCTTCAGTTC	GATTTTCAACCACTTCATGC	60	168
*HSD3B*	NM_000198	GGAAGCCAAGCAGAAAACCG	GCCCCTGTTGCCTTCTGTAT	58	172
*HSD11B1*	NM_005525	CTCTACAGAAGGTGGTATCC	AATGAGCATGTCTAGTCCTC	61	142
*HSD17B5*	NM_003739	TGAGGAGAAGCAGCAGCAAA	GGCGGAACCCAGCTTCTATT	60	190
*PGR*	NM_001202474	ATTCACTTTTTCACCAGGTC	AACCTGGCAATGATTTAGAC	60	180
*StAR*	NM_000349	GGCCTTGGGCATCCTTAG	TCCACCACGACCTCCAG	60	131
*TBP*	NM_003194	TGTGCTCACCCACCAACAAT	AGTCGTCTTCCTGAATCCCT	60	199

*AR*, androgen receptor; *CYP11A1*, cholesterol side-chain cleavage enzyme P450scc; *CYP19*, cytochrome P450 aromatase; *ERα* and *ERβ*, estrogen receptor alpha and beta; *GLUT4*, glucose transporter 4; *GPER*, G protein-coupled estrogen receptor 1; *GR*, glucocorticoid receptor; *HSD11B1*, 11beta-hydroxysteroid dehydrogenase 1; *HSD17b5*, 17beta-hydroxy steroid dehydrogenase 5; *HSD3B*, 3beta-hydroxysteroid dehydrogenase; *PGR*, progesterone receptor; TBP, TATA-box-binding protein.

### Hormone assays

Cell supernatants were collected and concentrations of the adipokines leptin (high-sensitive leptin ELISA, IBL, Hamburg, Germany) and adiponectin (Quantikine® ELISA Human Total Adiponectin/Acrp30, BioVendor, Kassel, Germany) were measured by ELISA according to manufacturer’s manual. ELISA data was normalized to the total protein concentration of individual sample. Protein was isolated using radioimmunoprecipitation assay (RIPA) buffer and the concentration was determined by the BioRad Protein Assay (BioRad). Furthermore, an ELISA kit (DRG Instruments, Marburg, Germany) was used for determining the concentration of cortisone.

Cholesterol and steroid hormones (cortisol, DHEAS, estradiol, progesterone, testosterone) were analyzed in the Central Laboratory of the University Hospital Halle. Cholesterol was measured using a colorimetric assay (CHOL2 Cholesterol Gen.2, Roche Diagnostics) on a Roche cobas c701 Analyzer integrated in a fully automated Roche Cobas 8000 platform. Cortisol, DHEAS, estradiol, progesterone and testosterone were measured with electrochemiluminescence Immunoassays (Elecsys Cortisol II, DHEA-S, Elecsys Progesterone III, Elecsys Testosterone II and Elecsys Estradiol III, Roche Diagnostics) on a Roche Cobas e602 Analyzer, also being part of the fully automated Roche Cobas 8000 platform. All analyses on the Roche Cobas Analyzers were carried out according to the manufacturer’s instructions and manuals, with routine maintenance and quality control procedures.

### LC-MS/MS

Steroids in SGBS cell supernatants were measured in the Department of Molecular Systems Biology of the Helmholtz-Centre for Environmental Research – UFZ Leipzig using the AbsoluteIDQ^®^ Stero17 Kit (Biocrates Life Sciences AG, Innsbruck, Austria). Sample preparation was performed by manufacturer’s instructions executed by a solid phase extraction procedure. The LC-MS/MS analyses were carried out by MRM acquisition (electrospray ionization, positive ionization mode) using a Waters Acquity UPLC System (Waters GmbH, Eschborn, Germany) coupled with Q-TRAP 5500 (Sciex, Darmstadt Germany GmbH). The quantification of LC-MS/MS generated data was performed by IntelliQuan algorithm from Analyst 1.6.2 software (Sciex) and evaluated by MetIDQ software (Biocrates).

### Immunohistochemistry for the localization of GLUT4

For the *in situ* immunodetection of GLUT4, SGBS adipocytes were cultured on chamber slides (Sarstedt, Nümbrecht, Germany). At day 8 cells were fixed with 4% paraformaldehyde for 25 min. After a washing step with PBS, endogenous peroxidases were blocked with H_2_O_2_ (3% in methanol) for 20 min. Further washing steps with tap water and PBST followed before the blocking with goat serum (10% in PBST) for 1 h. The primary antibody against GLUT4 (1:300; Sigma Aldrich) was incubated at 4°C overnight. Afterwards the secondary HRP-conjugated antibody goat anti-rabbit (1:1; Dako) was applied for 90 min at room temperature. Detection was performed with 3,3'-diaminobenzidin and hematoxylin was used for nuclear counterstaining.

### Triglyceride assay

The ‘Adipogenesis Kit’ (Sigma Aldrich) was used to detect the total cellular concentrations of triglycerides according to manufacturer’s manual. The coupled enzyme assay results in a colorimetric product, proportional to the trigylcerides present at day 8 of adipogenesis. Analyses were performed with the Synergy™ Mx microplate reader (BioTeK Inc., Winooski, VT, USA). Data were normalized to the protein concentration of each individual sample. Protein was isolated using radioimmunoprecipitation assay (RIPA) buffer and concentrations were determined by the BioRad Protein Assay (BioRad).

### Statistical analyses

At least four independent experiments (N) were performed with 12 wells pooled per treatment and experiment. Data were presented as mean ± s.e.m. Results of adipogenesis experiments were investigated by the ANOVA with the Bonferroni’s post hoc test in relation to the control group. To evaluate differences between the treatment with and without androstenedione (AD) an unpaired Student’s *t*-test or if necessary the Wilcoxon rank-sum test was used. Data differences were considered as statistically significant at *P* value ≤ 0.05.

## Results

### Androstenedione does not affect SGBS cell proliferation and differentiation

**Figure 1:**
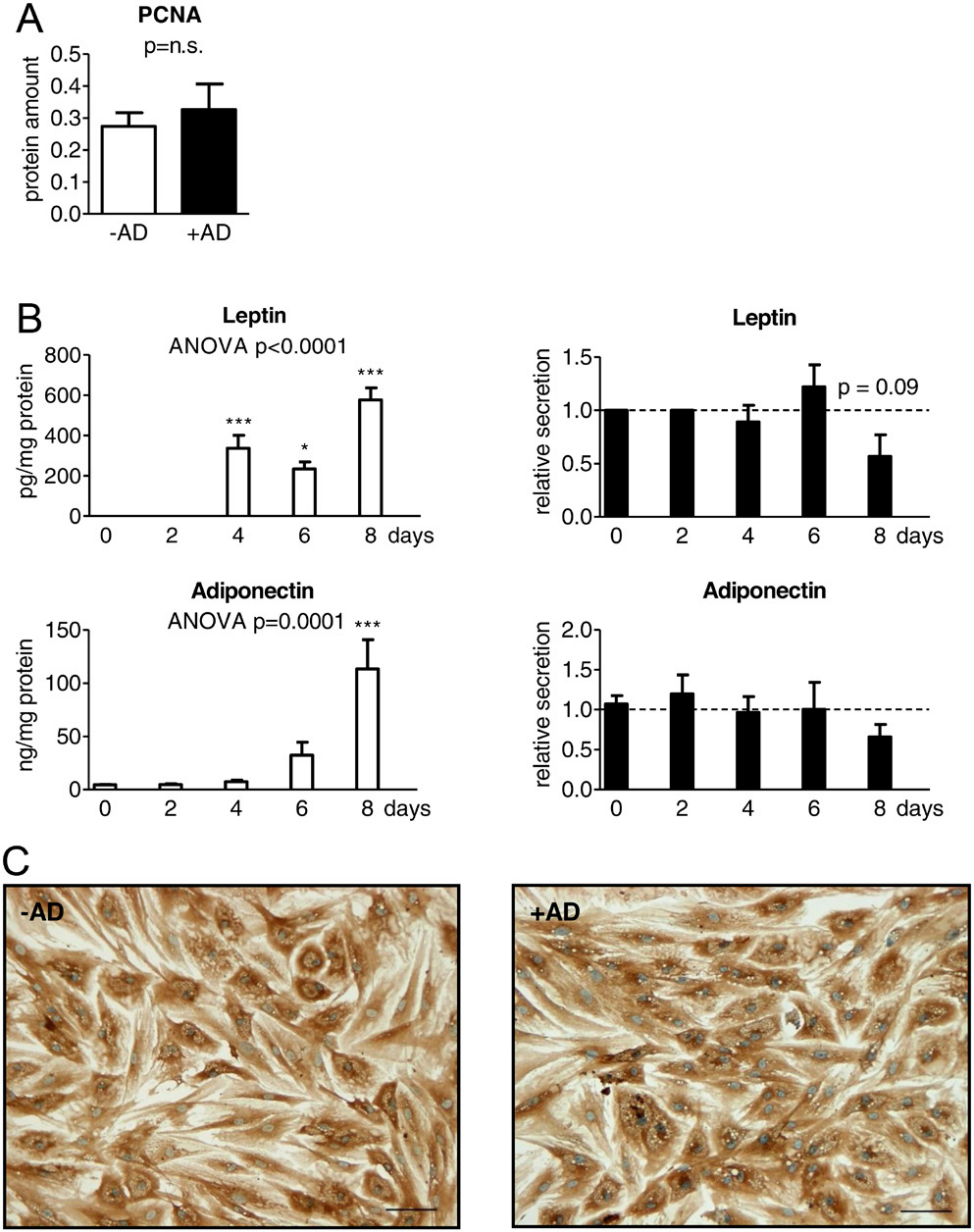
Influence of androstenedione on SGBS cell proliferation and differentiation. (A) SGBS cell proliferation was analyzed in SGBS preadipocytes before induction of adipogenic differentiation by determination of the amount of the proliferating-cell-nuclear-antigen (PCNA) under normal conditions and with application of androstenedione (AD) normalized to β-Actin (*n* = 4; *P* ≤ 0.05 ^#^ for comparing unexposed and AD-exposed cells). (B) For SGBS cell differentiation, secretion of leptin and adiponectin was measured during adipogenic differentiation under normal conditions (left). Additionally, AD stimulation is described in relation to the corresponding AD-free control level indicated as dotted line (right) (*n* = 4; * *P* ≤ 0.05; ** *P* ≤ 0.01; *** *P* ≤ 0.001 for normal adipogenesis in comparison to day 0 and^ #^ *P* ≤ 0.05; ^##^ *P* ≤ 0.01; ^###^ *P* ≤ 0.001 for comparing unexposed and AD-exposed cells). (C) For investigating the localization of GLUT4, immunodetection (brown staining) of this glucose transporter was performed *in-situ* of adult adipocytes (day 8) with nuclear counterstaining by hematoxylin (blue staining) comparing unexposed and AD exposed. The scale bar indicates 100 µM.

Cell proliferation of SGBS preadipocytes was investigated by determining the PCNA amount, but no influence of androstenedione was detectable (Fig. 1A). Furthermore, adipogenic differentiation was evaluated by measuring the secretion of the adipokines leptin and adiponectin in cell supernatants during adipogenesis (Fig. 1B). As expected, both adipokines increased during normal adipogenesis compared to day 0. Leptin was not detectable at day 0 and day 2, but significantly elevated at days 4, 6 and 8. The slight decline from day 4 to day 6 is not significant. With day 8 the highest level of leptin was measured reaching significant value also compared to day 4 and day 6. Adiponectin showed significantly elevated values at day 8 (up to the 25-fold compared to day 0) only. After the application of androstenedione the levels of both adipokines were not significantly different compared to the corresponding control samples of the same day (Fig. 1B). The investigation of several adipogenic and differentiation related genes such as *PPAR*s, *GLUT4*, *FABP4* and *CD36* revealed no effect on SGBS adipogenesis by androstenedione. Furthermore, we investigated the localization of GLUT4 within adult adipocytes (d8). Its inactive form is accumulated in the membrane of the Golgi apparatus. Due to an insulin-dependent activation the glucose transporter is translocated to the cell membrane. A positive staining was detectable in the cytoplasm of the adipocytes. However, the more intensely stained adipocyte cell lining recommends a predominant membrane-bound distribution that was not changed by androstenedione (Fig. 1C).

### Androstenedione causes accumulation of triglycerides

**Figure 2:**
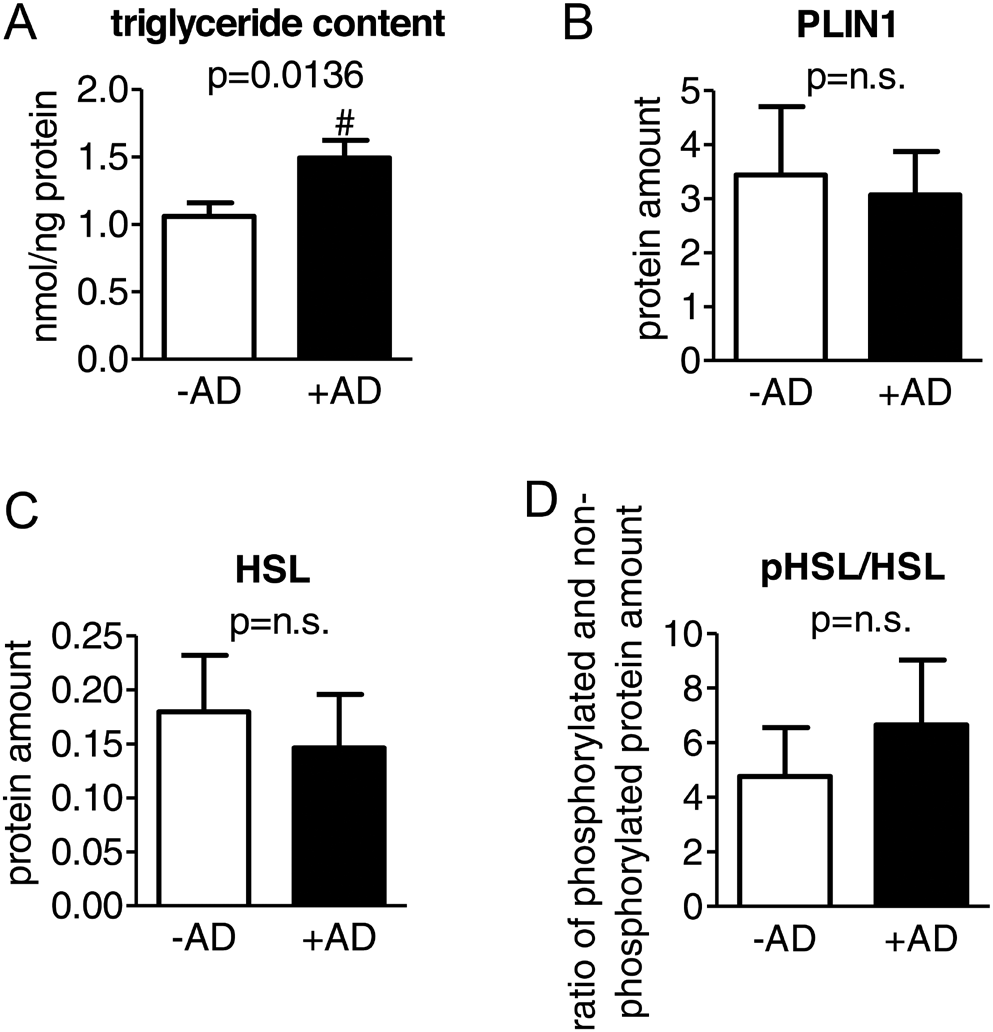
Influence of androstenedione on the lipid content within adult SGBS adipocytes. (A) The triglyceride content within adult SGBS adipocytes of day 8 was measured (*n* = 16; *P* ≤ 0.05 ^#^ for comparing ±AD). Furthermore, the protein amount of (B) perilipin (PLIN1) normalized to β-ACTIN and (C) HSL as well as (D) the activation of HSL by the quotient of the phosphorylated to the non-phosphorylated protein normalized to GAPDH were determined (*n* = 4).

The triglyceride content within the adult adipocytes (day 8) was measured revealing 1.4-fold higher triglyceride levels in the cells treated with androstenedione (Fig. 2A). Additionally, we analyzed the amount of PLIN1 and HSL as well as the activation of HSL by determining the quotient of the phosphorylated to the non-phosphorylated protein, but no alteration by androstenedione was detectable for both the protein amount and the activation (Fig. 2B, C and D).

### Androstenedione stimulates the secretion of progesterone, testosterone and cortisone

**Figure 3:**
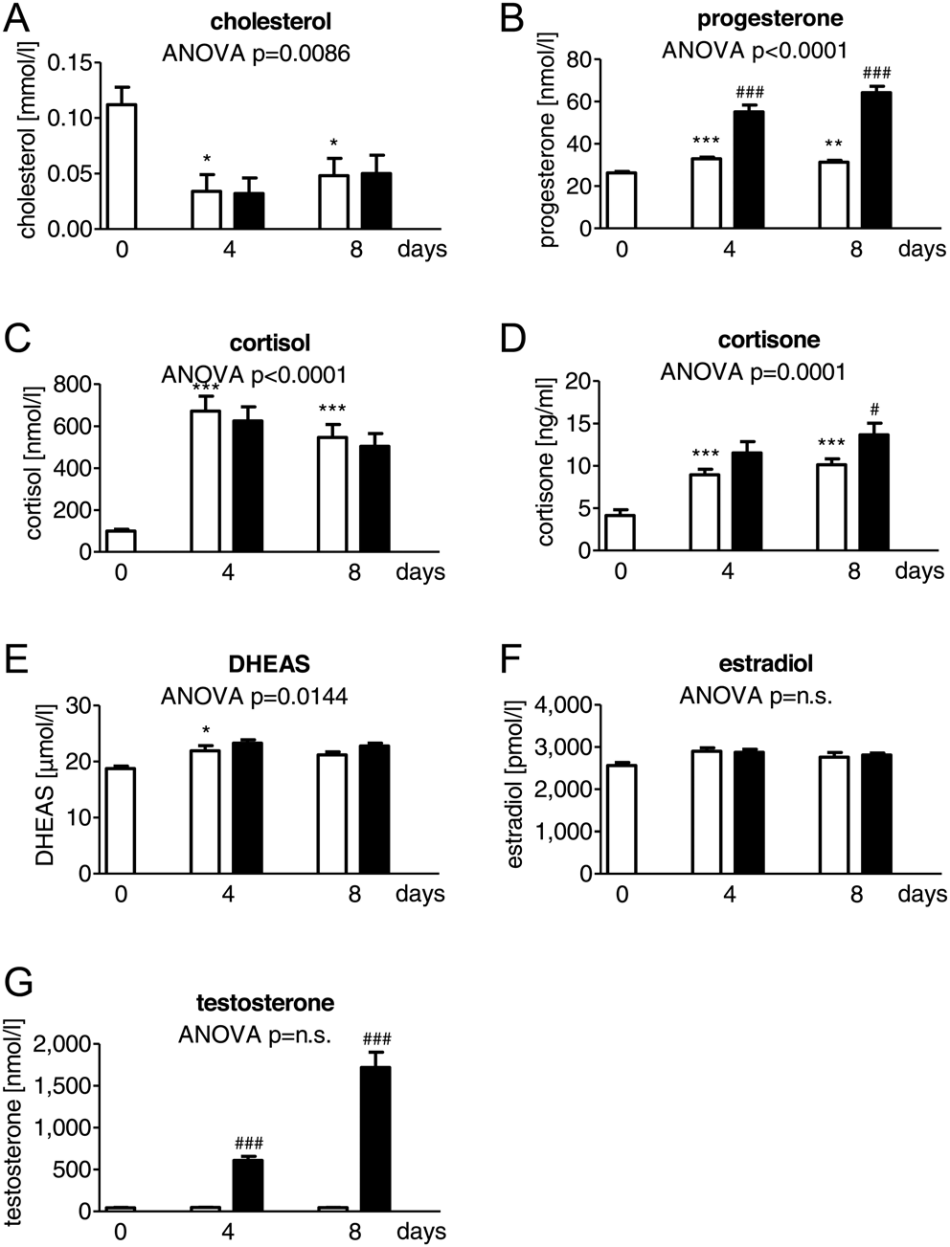
Influence of androstenedione on the steroid hormone synthesis in the SGBS cell model during adipogenesis. (A) Cholesterol, (B) progesterone, (C) cortisol, (D) cortisone, (E) dehydroepiandrosterone sulfate (DHEAS), (F) estradiol and (G) testosterone were determined in SGBS cells during adipogenic differentiation in controls (white columns) and with AD stimulation (black columns) (*n* = 5; * *P* ≤ 0.05; ** *P* ≤ 0.01; *** *P* ≤ 0.001 for normal adipogenesis in comparison to day 0 and^ #^ *P* ≤ 0.05; ^##^ *P* ≤ 0.01; ^###^ *P* ≤ 0.001 for comparing unexposed and AD-exposed cells).

Cholesterol and steroid hormone levels were determined during adipogenesis in supernatants of SGBS cells under normal conditions and after androstenedione-treatment with immunoassays (Fig. 3) and LC-MS/MS (Table 2). During normal adipogenesis cholesterol significantly decreased in the supernatant of SGBS cells at day 4 and day 8 (by 70% at day 4 and by 57% at day 8 each compared to day 0) (Fig. 3A). Simultaneously, a significantly increased secretion of progesterone (up to 1.3-fold), cortisol (up to 7-fold) and cortisone (up to 2-fold) was detectable at day 4 and day 8 compared to day 0 (Fig. 3B, C and D). In addition, DHEAS showed an up to 1.1-fold increase at day 4 compared to day 0 (Fig. 3E). Estradiol and testosterone did not change during adipogenesis (Fig. 3F and 3G). Application of androstenedione caused a significant increase of progesterone up to the 1.7-fold at day 4 and up to 2-fold at day 8 compared to the corresponding control sample of the same day (Fig. 3B). The same was observed for testosterone with a 13-fold increase at day 4 and up to 38-fold increase at day 8 compared to the corresponding control sample of the same day (Fig. 3G). For cortisone up to 1.4-fold increase compared to the control sample of the same day could only be observed at day 8 (Fig. 3D).

**Table 2:** Steroid hormone analysis performed by LC-MS/MS.

Hormone	Normal adipogenesis (µmol/L)			+AD (µmol/L) day 8
	day 0	day 4	day 8	
Aldosterone	0.005±0.002	0.032±0.007^a^	0.016±0.010	0.026±0.007
17alpha-hydroxyprogesterone	0.015±0.010	0.112±0.109	0.014±0.012	0.029±0.028
Androstenedione	4.957±1.260	13.918±2.968^a^	3.567±0.994	67.073±33.092
Androsterone	1.014±0.272	3.970±1.032^a^	0.847±0.219	158.753±13.614^f^
Corticosterone	0.005±0.003	0.291±0.037^c^	0.217±0.013^c^	0.219±0.023
Cortisol	0.301±0.155	34.853±1.926^c^	28.813±1.251^c^	28.813±1.585
Cortisone	0.080±0.019	2.112±0.137^b^	3.990±0.610^c^	8.770±0.659^f^
11-Deoxycorticosterone	0.013±0.013	0.578±0.366	0.091±0.068	0.013±0.008
11-Deoxycortisol	0.002±0.001	0.054±0.013^b^	0.015±0.002	0.054±0.006^f^
DHEA	0.080±0.080	0.160±0.090	0.861±0.428	0.033±0.021
Dihydrotestosterone	0.058±0.013	0.122±0.020^a^	0.056±0.010	6.689±1.106^f^
Estradiol	0.030±0.004	0.113±0.040	0.024±0.001	0.047±0.011
Estrone	0.760±0.701	0.176±0.049	0.099±0.036	0.046±0.011
Etiocholanolone	0.938±0.271	3.918±1.030^a^	0.832±0.219	158.673±13.619^f^
Progesterone	0.475±0.350	2.468±1.429	1.092±0.450	0.519±0.251
Testosterone	1.747±0.281	4.142±0.770^a^	1.260±0.270	99.833±21.772^e^

Data are presented as mean±s.e.m. (*n* = 5; ^a^*P* ≤ 0.05; ^b^*P* ≤ 0.01; ^c^*P* ≤ 0.001 for normal adipogenesis in comparison to day 0 and ^d^*P* ≤ 0.05; ^e^*P* ≤ 0.01; ^f^*P* ≤ 0.001 for comparing unexposed and androstenedione (AD)-exposed cells at day 8).

The above-mentioned findings were replicated by LC-MS/MS and additional steroid hormones were analyzed. Under normal conditions, androgens (androstenedione, androsterone, testosterone, dihydrotestosterone, etiocholanolone) and corticosteroids (corticosterone, aldosterone, 11-deoxycortisol, cortisol, cortisone) increased during adipogenesis. Furthermore, an androstenedione-mediated increase was found for androsterone, 11-deoxycortisol, dihydrotestosterone and etiocholanolone at day 8 (data are provided in Table 2).

### Androstenedione upregulates the enzyme for cortisol-cortisone interconversion

**Figure 4:**
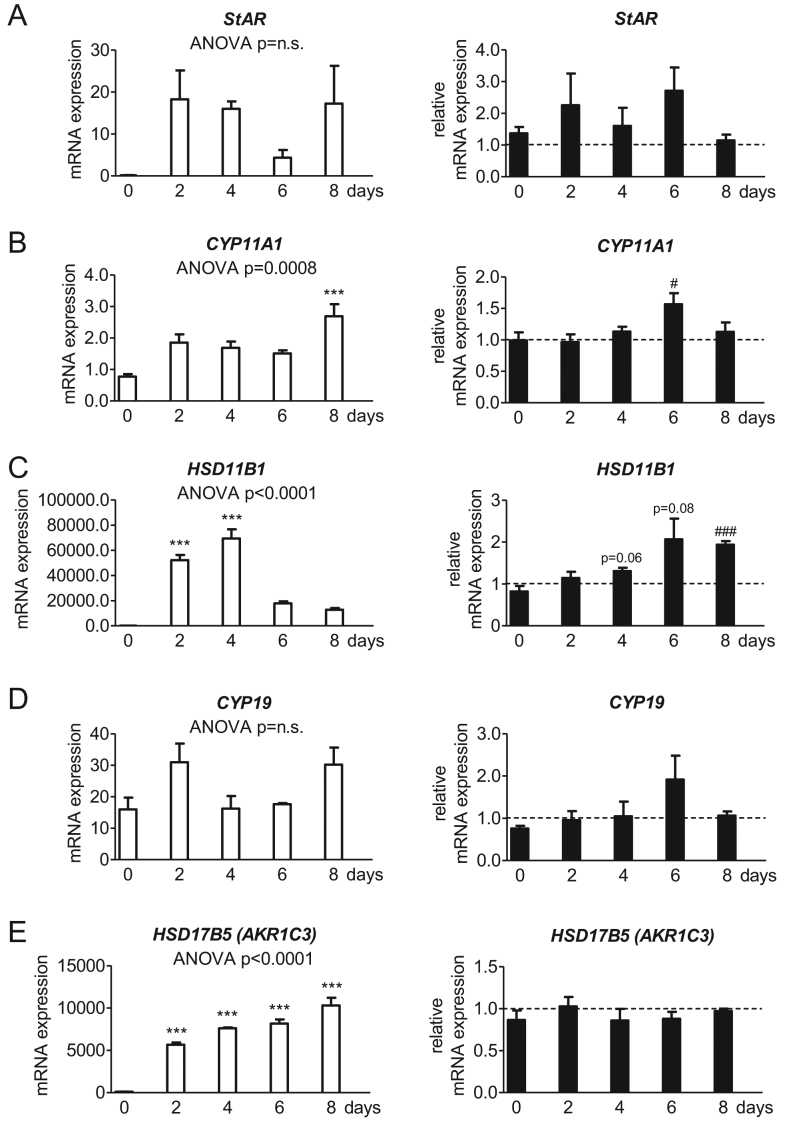
Gene expression of enzymes of the steroid hormone metabolism in the SGBS cell model during adipogenesis. The absolute mRNA expression of (A) the steroidogenic acute regulatory protein (*StAR*), (B) the cholesterol side-chain cleavage enzyme P450scc (*CYP11A1*), (C) 11beta-hydroxysteroid dehydrogenase 1 (*HSD11B1*), (D) the cytochrome P450 aromatase (*CYP19*) and (E) the aldo-keto reductase family 1 member C3 AKR1C3 (*HSD17B5*) normalized to 10^3^ molecules of *TBP* as housekeeping gene was evaluated in SGBS cells during adipogenic differentiation under normal conditions (left). Additionally, AD stimulation is described in relation to the corresponding AD-free control level indicated as dotted line (right) (*n* = 4; * *P* ≤ 0.05; ** *P* ≤ 0.01; *** *P* ≤ 0.001 for normal adipogenesis in comparison to day 0 and^ #^ *P* ≤ 0.05; ^##^ *P* ≤ 0.01; ^###^ *P* ≤ 0.001 for comparing unexposed and AD-exposed cells).

The transcription of enzymes important for the synthesis and the metabolism of steroid hormones were investigated under normal conditions and after androstenedione treatment. SGBS cells expressed the machinery of the *de novo* steroid biosynthesis from cholesterol, namely *StAR* (Fig. 4A) and *CYP11A1* (Fig. 4B). Furthermore, SGBS cells expressed* HSD11B1* for the cortisol-cortisone interconversion (Fig. 4C), the aromatase *CYP19* (Fig. 4D) and the enzyme for the androgen interconversion *HSD17B5* (Fig. 4E). *StAR* (Fig. 4A) and *CYP19* (Fig. 4D) showed no alterations compared to day 0 during normal adipogenesis. However, significant changes were observed with increasing transcription for *CYP11A1* at day 8 (up to the 3-fold at day 8 compared to day 0; Fig. 4B), *HSD11B1* at day 2 and day 4 (up to the 356-fold at day 2 and up to the 474-fold at day 4 each compared to day 0; Fig. 4C) and *HSD17B5* at every measuring point (up to the 54-fold at day 2, up to the 73-fold at day 4, up to the 78-fold at day 6 and up to the 99-fold at day 8 each compared to day 0; Fig. 4E). *CYP17A1* and *HSD3B*, both metabolizing pregnenolone to downstream steroids, were detectable by sequencing, but the expression was too low for quantification by qRT.

Androstenedione treatment only significantly affected the expression of *CYP11A1* at day 6 (up to the 1.6-fold compared to the corresponding control sample of the same day; Fig. 4B) and *HSD11B1* at day 8 (up to the 1.9-fold compared to the corresponding control sample of the same day; Fig. 4C).

### Androstenedione lowers *ERβ* and *AR* transcription

**Figure 5:**
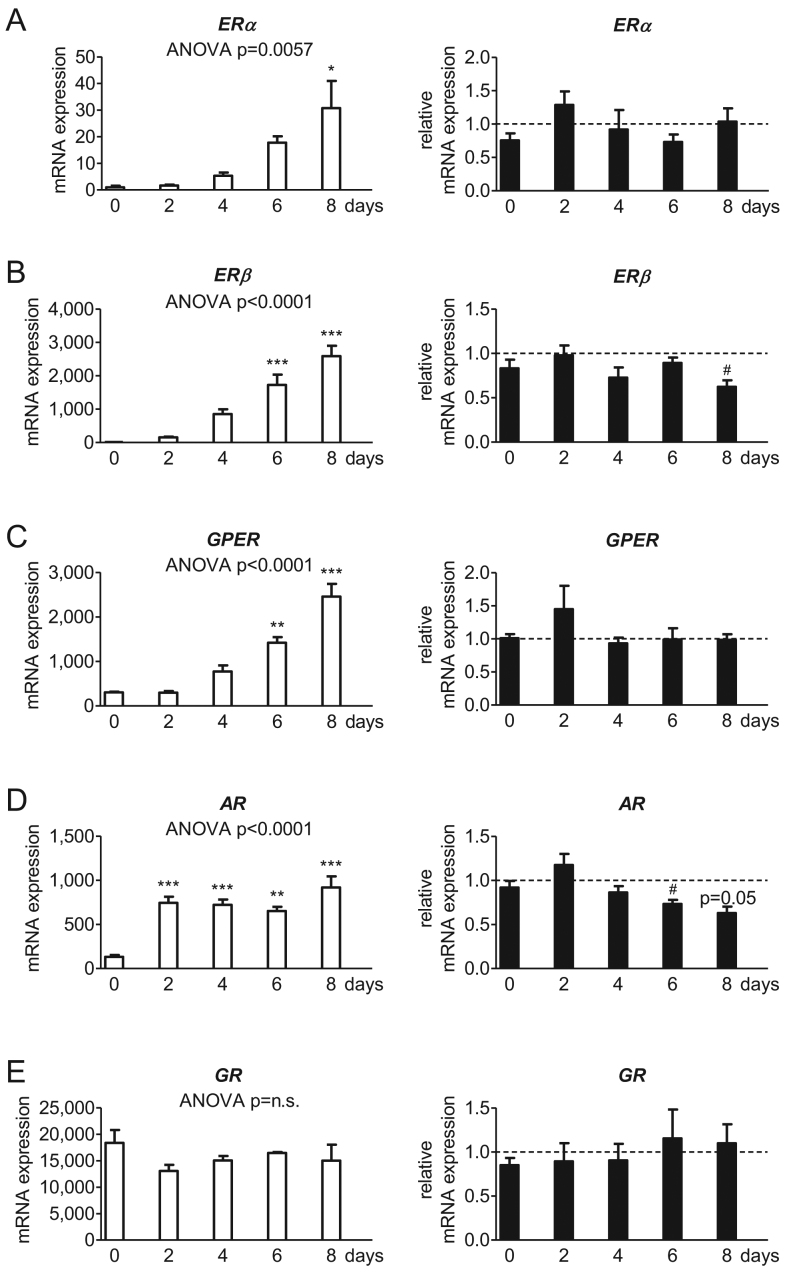
Influence of androstenedione on estrogen receptors in the SGBS cell model. The absolute mRNA expression of (A) estrogen receptor alpha (*ERα*) and (B) beta (*ERβ*) as well as (C) the G protein-coupled estrogen receptor 1 *(GPER*), (D) the androgen receptor (*AR*) and (E) the glucocorticoid receptor (GR) normalized to 10^3^ molecules of *TBP* as housekeeping gene was evaluated in SGBS cells during adipogenic differentiation under normal conditions (left). Additionally, AD stimulation is described in relation to the corresponding AD-free control level indicated as dotted line (right) (*n* = 4; * *P* ≤ 0.05; ** *P* ≤ 0.01; *** *P* ≤ 0.001 for normal adipogenesis in comparison to day 0 and^ #^ *P* ≤ 0.05; ^##^ *P* ≤ 0.01; ^###^ *P* ≤ 0.001 for comparing unexposed and AD-exposed cells).

Gene expression of estrogen receptors, the androgen receptor, the progesterone receptor and the glucocorticoid receptor were studied. For estrogen and androgen receptors, we found an upregulation during normal adipogenesis: significant values were reached for *ERα* at day 8 (up to the 24-fold at day 8 compared to day 0; Fig. 5A), *ERβ* and *GPER* both at day 6 and day 8 (*ERβ*: up to the 135-fold at day 6 and up to the 202-fold at day 8 each compared to day 0; Fig. 5B; *GPER*: up to the 5-fold at day 6 and up to the 8-fold at day 8 each compared to day 0; Fig. 5C) as well as *AR* at every measuring point (up to the 6-fold at day 2, up to the 5-fold at day 4, up to the 5-fold at day 6 and up to the 7-fold at day 8 each compared to day 0; Fig. 5D). Employment of androstenedione significantly lowered the transcription of *ERβ* at day 8 (by 41% compared to the corresponding control sample of the same day; Fig. 5B) and *AR* at day 6 and day 8 (by 27% at day 6 and by 37% at day 8 compared to the corresponding control sample of the same day; Fig. 5D). The glucocorticoid receptor was expressed, too, without showing an alteration during normal adipogenesis or after androstenedione application (Fig. 5E). The progesterone receptor was detectable by sequencing, but the expression was too low for quantification by qRT.

## Discussion

Adipose tissue has been recognized as an steroidogenic organ producing and transforming a wide range of steroid hormones in a complex network of steroidogenic enzymes (22, 23, 24). The ability of *de novo* synthesis of steroid hormones from cholesterol depends on the presence of the mitochondrial cholesterol transport machinery and CYP11A1 that converts cholesterol into pregnenolone. In our study, *StAR* – one major component of the mitochondrial cholesterol transport machinery – and *CYP11A1* only were present with increasing expression of *CYP11A1* during SGBS adipogenesis. Simultaneously, cholesterol level in supernatants of maturating adipocytes decreased, and several steroid hormones were detectable, namely progesterone, androgens, estradiol and corticosteroids. Estrogen as well as the *CYP19* expression was not altered during adipogenesis. Data from SGBS over an 8–21-day differentiation period showed a steady decrease of *CYP19* (31), and the comparison of preadipocyte and adult adipocyte from primary human culture revealed a significant downregulation (32). Furthermore, we found estrogen receptors to be expressed in SGBS cells. Preadipocytes had only low copies of *ERα *, *ERβ* and *GPER* with a significant increase during differentiation. The occurrence of estrogen receptors and their up-regulation during adipogenesis has already been described in SGBS and 3T3-L1 cells, assuming an involvement in the differentiation of preadipocytes (33, 34). An elevation of the androgen level was found solely at day 4, whereas for the androgen interconverting* HSD17B5* until day 8. This is in agreement with data of differentiating stromal preadipocytes from s.c. fat biopsies to mature adipocytes (35). As expected, we found the *AR* to be expressed in maturing SGBS cells with a significant increase at all analysed time points. AR has already been demonstrated to be present in human adipocytes and SGBS cells (33, 36, 37). An upregulated transcription but down-regulated activity of AR as a result of GR-activation is an essential step for inducing adipocyte differentiation driven by dexamethasone, an important supplement in the culture media used during adipogenesis (25, 26, 38, 39). Interestingly, *GR* expression did not change, which may have resulted from an already reached maximum induction by the culture media additive dexamethasone. At glucocorticoid level, corticosterone and both cortisol and cortisone, increased during overall normal maturation. Despite the fact of cortisol-supplementation in the culture media during adipogenesis (25, 26), SGBS cells secreted cortisol by themselves that displayed an up to 7-fold (day 4) and 6-fold (day 8) rise when compared to values at day 0. *HSD11B1,* a bidirectional cortisol-cortisone-interconverting enzyme (40), was upregulated only during induction stage. During 3T3-L1 adipogenesis *HSD11B1* increased and an activation of expression was paralleled by human adipocyte differentiation (27, 40). On the base of these results we suggest, that SGBS adipocytes have a functional *de novo* steroid hormone biosynthesis and are a suitable steroidogenic fat cell model to simulate diverse pathological scenarios.

Investigating the hypothesis of a substantial contribution to androgen synthesis by adipose tissue (10), we treated maturing SGBS preadipocytes with an exceeding concentration of androstenedione, the precursor of active steroid hormones. First, androstenedione did not affect proliferation of cells or the secretion of leptin and adiponectin. Both adipokines were taken as validation markers for a correct maturation of the SGBS preadipocytes as previously described (26). Studies on the SGBS model and primary human s.c. adipocytes with active androgens demonstrated no significant influence on proliferation, but a negative impact on differentiation through an AR-driven pathway reducing leptin but not adiponectin secretion (33, 37, 41, 42, 43). Interestingly, the precursor dehydroepiandrosterone impaired cell proliferation without significant effect on differentiation (44). Analyses of the insulin-regulated glucose transporter GLUT4 revealed an upregulated gene expression (data not shown) and a predominant membrane-bound distribution under normal conditions, reasoned by the association of adipogenesis with increasing insulin-sensitivity (45, 46). However, androstenedione had no impact on GLUT4. Treatment of the human s.c. cell line Chub7 with dehydroepiandrosterone caused an elevation of the basal, but not insulin-stimulated glucose-uptake (47). Quantification of the lipid accumulation in SGBS revealed a 40% elevated triglyceride content within the adult adipocytes under androstenedione treatment. In this regard, we found no influence of androstenedione on PLIN1 and HSL (components of the lipid droplets). In contrast to our results, potent androgens decreased lipid content in the same cell model and in primary human adipocytes (37, 43). Another study demonstrated no alteration of the basal but reduced cAMP-stimulated lipolysis with no effect on PLIN1 (48). PLIN1 is a highly expressed adipocyte-specific protein on the surface of lipid vesicles associated with the stored triglyceride content. Thereby, it contributes to homeostasis in lipid metabolism by blocking basal and mediating hormone-stimulated lipolysis (49, 50, 51, 52).

Second, as the cholesterol level did not change, we assume the employed androstenedione did not activate the *de novo* steroid hormone synthesis, but rather was converted into other steroids. The secretion of progesterone, testosterone (additionally dihydrotestosterone as well as the less active androgenic metabolites androsterone and etiocholanolone) and cortisone was accelerated by androstenedione. A new finding is the androstenedione-mediated induction of the progesterone level as well as of the *CYP11A1* expression in adipocytes. Interestingly, granulosa cells synthesize significantly higher levels of estrone and estradiol without changes of progesterone and androgen under low concentrations of androgen precursors with the limitation that higher concentrations would lead to increasing androgen levels by possibly masking the precursor effect (53). However, the treatment of granulosa cells with a potent androgen accelerates progesterone synthesis and enhances the expression of *StAR* and *CYP11A1*, but otherwise had no significant effect on estrogen synthesis (54). Downstream of progesterone to glucocorticoids we found alterations as followed: 11-deoxycortisol and furthermore, cortisone – but not cortisol – were elevated at day 8. In this regard, *HSD11B1* was changed in the same way. *HSD11B1*-expression was found to be increased in *in vitro* androgen-treated male preadipocytes (32). Furthermore, *HSD11B1* expression was positively correlated in s.c. fat of obese women with circulating testosterone (55). As HSD11B1 is responsible for the bidirectional cortisol-cortisone-interconversion, in adipose tissue the dehydrogenase activity (cortisol to cortisone) predominates the oxidoreductase activity (cortisone to cortisol). Accordingly, this predominance stood against previous assumption of a linear relation between the intra-adipose glucocorticoid and HSD11B1 level (56). In Chub-S7 preadipocytes, the *HSD11B1* expression was reduced by dehydroepiandrosterone. Simultaneously, its enzyme activity revealed a decreased oxidoreductase activity with concurrent increased dehydrogenase activity (47). The switch to the predominant conversion to hormonally inactive glucocorticoids implicates a metabolic protective mechanism (57).

Summing up, the present study describes a functional *de novo* steroid hormone biosynthesis for the SGBS adipogenic cell model. Thereby, our results confirm the steroidogenic activity of adipocytes and the function of adipose tissue as a steroidogenic organ. Treatment with the androgen precursor androstenedione had no influence on the adipogenic differentiation to mature SGBS adipocytes and did not cause lipolytic effects. However, androstenedione application significantly altered steroidogenesis: to our knowledge, we are the first to show an induction of progesterone production in adipocytes. Besides this, downstream steroid hormone secretion points towards an upregulated androgen and glucocorticoid pathway, whereas the estrogenic pathway seemed not to be affected. Our current data suggest that adipocytes do not initiate androgen excess, but contribute to an already persisting androgen excess.

Despite a multitude of advantages, *in vitro* cell models like SGBS are also subject to limitations. One obvious limitation is the lack of systemic crosstalk, like hormonal interactions, which occur in a whole organism. Whether differentiation and proliferation processes in cell models mimic the precise course *in vivo* seems difficult to assess. Furthermore, the obtained results in single cell cultures cannot directly be transferred to *in vivo* situation. Nevertheless, the SGBS model is clearly closer to other human systems than murine models. The SGBS model allows identifying the main players and central signaling pathways with their regulations.

## Declaration of interest

The authors declare that there is no conflict of interest that could be perceived as prejudicing the impartiality of the research reported.

## Funding

Jana Ernst and Kristina Schädlich were supported by the Roux Programme of the Faculty of Medicine, Martin Luther University Halle-Wittenberg (JE FKZ 29/11, KS FKZ 26/06). We acknowledge the financial support within the funding programme Open Access Publishing by the German Research Foundation (DFG).

## Author contribution statement

J E: acquisition, analysis and interpretation of data, conception and design of the study, drafting the article and final approval. K G: acquisition, analysis and interpretation of data, final approval. F B K: acquisition, analysis and interpretation of data, revising the article and final approval. U E R-K: analysis of data, final approval. M W: contribution to the technique of SGBS cell culture, discussion of the data, final approval. F D: project leader, revising the article and final approval. K S: project leader, conception and design of the study, revising the article and final approval.

## Acknowledgements

The authors thank Christine Froehlich for excellent technical assistance.
